# COVID‐19 reinfection or reactivation in a renal transplant patient

**DOI:** 10.1002/ccr3.4672

**Published:** 2021-08-16

**Authors:** Sara Abolghasemi, Farnaz Zolfaghari, Mohammad Naeimipoor, Hamed Azhdari Tehrani, Atousa Hakamifard

**Affiliations:** ^1^ Infectious Diseases and Tropical Medicine Research Center Shahid Beheshti University of Medical Sciences Tehran Iran; ^2^ Department of Hematology and Medical Oncology Shahid Beheshti University of Medical Sciences Tehran Iran

**Keywords:** COVID‐19, immunity, immunocompromised host, kidney transplantation, recrudescence, recurrence, relapse, SARS‐CoV‐2

## Abstract

Recurrences of COVID‐19 infection may occur in immunocompromised patients. Reinfection or reactivation of COVID‐19 virus is a challenging issue in these patients.

## Graphical abstract

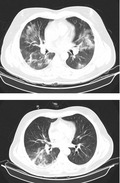

## 1 INTRODUCTION

We present a case of recurrent COVID‐19 pneumonia who underwent living‐related renal transplantation 10 years ago. Returning of symptomatic immunocompromised patients who had a history of recovered COVID‐19 disease with a positive real‐time reverse transcriptase‐polymerase chain reaction (rRT‐PCR) test after weeks to months of the previous episode is a challenging issue.

The novel coronavirus severe acute respiratory syndrome coronavirus‐2 (SARS‐CoV‐2) was first identified toward the end of 2019 in Wuhan, Hubei Province of China, as the cause of a new viral respiratory illness.^1^ Experience in the management of COVID‐19 in the post‐transplant population is limited. Due to its high infectivity, transplant patients are vulnerable, especially those with comorbidities. Post‐transplant patients are often under immunosuppressive therapy; this immune deficiency state results in opportunistic infections. In the case of COVID‐19 infection, these patients are particularly at risk of severe complications due to their immunosuppression and coexistence of comorbidities.^2^, ^3^, ^4^ Here, we present a case of renal transplant patient with recurrent COVID‐19 pneumonia. Returning of symptomatic immunocompromised patients who had a history of recovered COVID‐19 disease with a positive real‐time reverse transcriptase‐polymerase chain reaction (rRT‐PCR) test after weeks to months of the previous episode and also differentiation between reinfection and reactivation is a challenging issue.

## 2 CASE PRESENTATION

**FIGURE 1:**
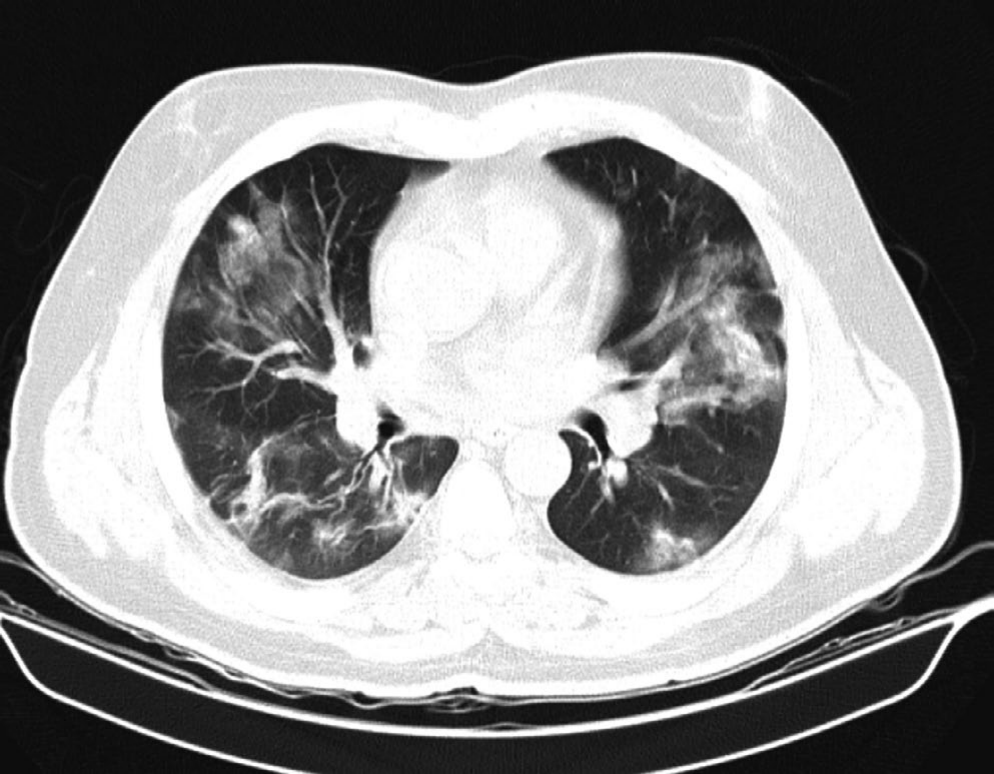
Chest CT scan revealed multifocal peribronchial ground‐glass opacities and consolidation at first admission

In August 2020, a 52‐year‐old man with a history of hypertension and ischemic heart disease and atrial fibrillation who underwent living‐related renal transplantation 10 years ago was presented with fever, dry cough, dyspnea, and oxygen saturation (SpO_2_) of 89% on room air. Drug history was as follows: apixaban, atorvastatin, carvedilol, losartan, nitrocontin, and spironolactone. Maintenance immunosuppressive drugs consisted of prednisolone 5 mg/d, cyclosporine 50 mg every 12 hours, and mycophenolate mofetil 500 mg every 12 hours. Creatinine base was 1.7 mg/dl. Vital signs at admission were as follows: pulse rate: 96 beats/ minute, respiratory rate: 22 breaths/ minute, and blood pressure: 110/70 mmHg. rRT‐PCR on samples obtained by oropharyngeal and nasopharyngeal swabs confirmed SARS‐CoV‐2 infection. Chest computed tomography scan was suggestive of COVID‐19–induced pneumonia [Figure 1].

Supportive oxygen therapy with the nasal cannula at 3–4 liters per minute, and treatment with remdesivir 200 mg on day 1 and 100 mg once daily for five days were started. In addition, the mycophenolate mofetil was discontinued and cyclosporine 50 mg every 12 hours was continued. Also, the dose of prednisolone was increased to 15 mg/d. On day 4, the SpO2 was 90% with relative improvement in dyspnea. On day 9, SpO2 was 94% on room air. Supportive oxygen therapy was discontinued. On day 10, the patient was discharged. After discharge, cyclosporine 50 mg every 12 hours was continued. Also, prednisolone was continued at a dose of 15 mg/d and then reduced to 5 mg/d within three weeks. When the prednisolone dose reached 5 mg, mycophenolate mofetil 500 mg every 12 hours was started again.

**FIGURE 2:**
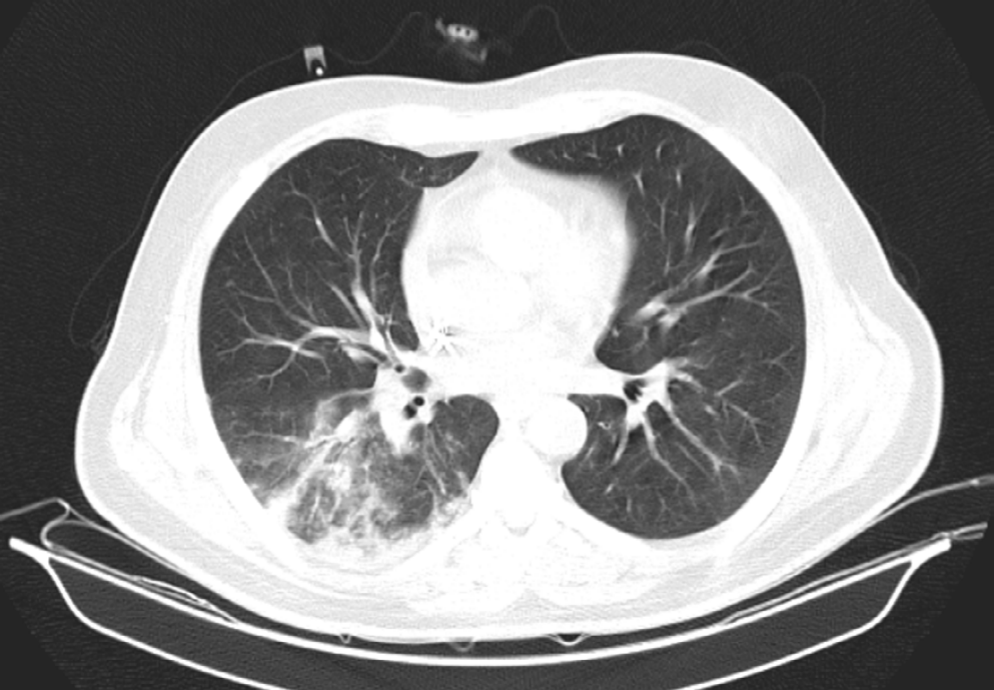
Chest CT scan revealed multifocal peribronchial ground‐glass opacities with consolidation in superior segment of RLL at second admission

47 days after discharge, the patient with seven‐days history of progressive dry cough, fever, dyspnea, myalgia, vomiting, and weakness was presented to emergency department. His vital signs at admission were as follows: pulse rate 125 beats/ minute, BP: 106/80, T: 38.6, and respiratory rate: 26 breaths/ minute. SpO_2_ was 86% on room air. rRT‐PCR on samples obtained by oropharyngeal and nasopharyngeal swabs was positive for SARS‐CoV‐2 infection again. High‐resolution chest CTs were obtained at admission [Figure 2], and supplemental oxygen was administered through a nasal cannula at a rate of 5 liters per minute. Treatment with remdesivir 200 mg on day 1 and 100 mg once daily for five days and dexamethasone 8 mg/day was started. Mycophenolate mofetil was discontinued again, and cyclosporine 50 mg every 12 hours was continued. On day 3, the patient was afebrile and SpO2 was 90% with improvement in dyspnea. On day 6, SpO2 was 92% on room air. The patient's condition improved over 12 days, and the patient was discharged with SpO2 = 96%, afebrile, and improved cough with resolution of dyspnea. After discharge, cyclosporine 50 mg every 12 hours was continued. Also, prednisolone was continued at a dose of 15 mg/d and then reduced to 5 mg/d within three weeks. When the prednisolone dose reached 5 mg, mycophenolate mofetil 500 mg every 12 hours was started again. Table 1 shows laboratory data, and Table 2 shows immunosuppressive drugs and clinical characteristics of the patient in 2 episodes.

**TABLE 1:** Laboratory data in 2 episodes and at discharge

Laboratory test	First episode	At discharge	Second episode	At discharge
WBC(/µl) Lymphocyte (/µl) PLT(/µl) Hb (gr/dl) BUN (mg/dl) Cr (mg/dl) AST (IU/L) ALT (IU/L) CRP (mg/l)	4500 540 398000 10 54 2.1 14 20 78	5100 920 321000 9.3 49 1.8 19 31 11	6800 816 343000 11.3 43 1.7 9 12 47	7200 1150 310000 11 31 1.6 20 10 7

WBC, white blood cells; CRP, C‐reactive protein; PLT, platelet; Hb, hemoglobin; BUN, blood urea nitrogen; Cr, creatinine; ALT, alanine aminotransferase; AST, aspartate aminotransferase.

**TABLE 2:** Immunosuppressive drugs and clinical characteristics of patient in two episodes

	First episode	Second episode
Symptoms at onset	Dry cough, dyspnea, fever	Dry cough, fever, dyspnea, myalgia, vomiting, and weakness
Maintenance immunosuppression before admission	Prednisolone 5 mg/d Cyclosporine 50 mg every 12 hours Mycophenolate mofetil 500 mg every 12 hours	Prednisolone 5 mg/d Cyclosporine 50 mg every 12 hours Mycophenolate mofetil 500 mg every 12 hours
Immunosuppressive drugs after confirmation of COVID−19 pneumonia	Prednisolone 15 mg/day Mycophenolate mofetil was discontinued Cyclosporine 50 mg twice daily	Dexamethasone 8 mg/d Mycophenolate mofetil was discontinued Cyclosporine 50 mg twice daily
Antiviral therapy	Remdesivir	Remdesivir
SpO_2_ on room air	89%	86%
Duration of hospitalization	10 days	12 days

## 3 DISCUSSION

We reported the clinical features and therapeutic course of a renal transplant recipient who experienced two episodes of COVID‐19 pneumonia. Till now, cases of renal transplant patients with COVID‐19 pneumonia are reported. Transplant recipients are at significant risk for COVID‐19 infection. The immunocompromised state predisposes these patients to greater susceptibility to infection, more rapid progression to pneumonia, and more disease severity.^5^ Regarding the case discussed in this report, clinical and radiological characteristics of COVID‐19 pneumonia were similar to those of other non‐transplanted adult patients with COVID‐19 pneumonia. However, the clinical manifestations in post‐transplant patients may be atypical.^6^

Management of immunosuppression in transplant recipients with COVID‐19 infection controversies due to the uncertain effects of immunosuppression on host viral defense and inflammatory response is reported. In general regarding treatment, there is a need to consider adjusting immunosuppressive agents while protecting graft function.^7^ In the cohort study by Chen et al. in 30 renal transplant patients, experience of withholding calcineurin inhibitors (CNIs) and antimetabolite during the management of COVID‐19 pneumonia did not increase the risk of allograft rejection.^8^ In the study reported by Zhu et al., a total of 10 renal transplant recipients were evaluated and the use of CNIs in seven patients was completely halted for about 9 days, and the dosage was halved for one patient for 12 days, but none of the eight patients developed acute renal graft rejection.^9^ There is evidence that mycophenolate and SARS‐CoV‐2 may exert a synergic effect on depleting peripheral lymphocytes, which can be responsible for an aberrant immune reconstitution.^10^ It is suggested that in mycophenolate‐containing immunosuppression regimen, mycophenolate was withdrawn with remaining other immunosuppressive drugs unchanged or with mild reduction.^11^ In the treatment of COVID‐19 pneumonia, steroid use has emerged as a supportive treatment for COVID‐19 pneumonia in guidelines, especially in severe cases.^12^ In a report by Fontana et al., a case of renal transplant with COVID‐19 pneumonia was treated with tocilizumab and hydroxychloroquine. Maintenance immunosuppression consisted of cyclosporine plus steroid. At first in the management of treatment, cyclosporine dose was reduced by a half, and then, considering the lack of improvement in clinical conditions this drug was withdrawn. Also, oral steroid dose was increased.^13^ In the study by Karatas et al. in renal transplant patients, mycophenolate mofetil, mycophenolic acid, or azathioprine was stopped and CNIs were interrupted. High‐dose methylprednisolone with or without initial pulse treatment was administered according to the severity of the disease.^14^ In the presented case, we discontinued mycophenolate, but cyclosporine and steroid were continued.

Another important entity is the recurrences of COVID‐19 infection as viral reinfection or reactivation. Our case was hospitalized for two times with clinical and radiological features compatible with COVID‐19 pneumonia. At first admission, the PCR test was positive. After 47 days, the patient was symptomatic again and the PCR test was positive.

There is some evidence that immunocompromised patients have prolonged viral shedding.^15^ The main point is that the PCR test cannot differentiate between infectious and non‐infectious viruses and viable and nonviable viruses, so not all test positives will be a clinical recurrence. Also, there is evidence that shedding of viable virus in patients with profound immunosuppression after undergoing hematopoietic stem cell transplantation or receiving cellular therapies may last for at least 2 months.^16^ In patients that have respiratory symptoms after one episode and have positive PCR test, recurrences should be differentiated from COVID‐19 complications such as pulmonary embolism or superinfection. Also, the persistence of traces of viral RNA that can be detected in respiratory samples up to 6 weeks after onset of symptoms in clinically cured patients should be considered. Pulmonary embolism and clues for superinfection and other differential diagnosis were ruled out in our case; hence in the presented case, episode of symptom recurrence after the first episode was attributed to COVID‐19 infection, but we cannot differentiate between reinfection and reactivation due to lack of access to the viral genome sequencing test. We did not perform the PCR test at discharge. As stated in the studies, the PCR test is not necessary at the time of discharge. In addition, if the test reported negative, it may be attributed to false‐negative causes and cannot be judged. The presented patient became symptomatic in two episodes 47 days apart with positive findings on CT scan and positive rRT‐PCR test. In both episodes, we manage the disease course with corticosteroid and remdesivir. Cyclosporine was continued, and mycophenolate mofetil was stopped during our management; the patient was recovered eventually with resolution of signs and symptoms. Immunosuppressive factors could contribute to impair viral clearance and led to SARS‐CoV‐2 reactivation.^17^ In the study by Gousseff et al., it was hypothesized that in the case of healthy patients with mild symptoms at both episodes recurrence due to the prolonged exposition can be supposed, but in severe cases (eg, hypoxemic pneumonia), recurrence might have occurred due to a suboptimal control of the SARS‐CoV‐2 infection, allowing a second episode of viral replication.^18^

## 4 CONCLUSION

Transplant recipient patients are particularly at risk of greater severity of COVID‐19 infection and complications due to their immunosuppression and coexistence of comorbidities. Recurrences of COVID‐19 infection may occur in these patients, and differentiation between reinfection and reactivation is a challenging issue.

## CONFLICT OF INTEREST

The authors declare that they have no competing interest.

## AUTHOR CONTRIBUTIONS

S.A, F.Z, M.N, H.AT, and A.H acquired data, and analyzed and interpreted the data. A.H wrote the first draft of the manuscript. S.A revised the manuscript. All authors have read and approved the final manuscript.

## ETHICAL APPROVAL

This research was approved by the ethics committee of Shahid Beheshti University of Medical Sciences (Ethical code: IR.SBMU.MSP.REC.1400.040). Written informed consent was obtained from the patient.

## Data Availability

Data sharing is not applicable to this case report type article as no new data were created or analyzed in this study.
